# IFNγ-Stimulated Dendritic Cell Exosomes for Treatment of Migraine Modeled Using Spreading Depression

**DOI:** 10.3389/fnins.2019.00942

**Published:** 2019-09-03

**Authors:** Kae M. Pusic, Lisa Won, Richard P. Kraig, Aya D. Pusic

**Affiliations:** Department of Neurology, The University of Chicago, Chicago, IL, United States

**Keywords:** exosomes, dendritic cells, interferon gamma, environmental enrichment, oxidative stress, migraine, spreading depression

## Abstract

Migraine is a common headache disorder characterized by unilateral, intense headaches. In migraine with aura, the painful headache is preceded by focal neurological symptoms that can be visual, sensory, or motor in nature. Spreading depression (the most likely cause of migraine with aura and perhaps related headache pain) results in increased neuronal excitability and related increases in inflammation and production of reactive oxygen species. This in turn can promote the transformation of low-frequency, episodic migraine into higher-frequency and eventually chronic migraine. Though migraine affects 11% of adults worldwide, with 3% experiencing chronic headache, existing therapies offer only modest benefits. Here, we focus on the mechanisms by which environmental enrichment (i.e., volitionally increased intellectual, social, and physical activity) mitigates spreading depression. In prior work, we have shown that exposure to environmental enrichment reduces susceptibility to spreading depression in rats. This protective effect is at least in part due to environmental enrichment-mediated changes in the character of serum exosomes produced by circulating immune cells. We went on to show that environmental enrichment-mimetic exosomes can be produced by stimulating dendritic cells with low levels of interferon gamma (a cytokine that is phasically increased during environmental enrichment). Interferon gamma-stimulated dendritic cell exosomes (IFNγ-DC-Exos) significantly improve myelination and reduce oxidative stress when applied to hippocampal slice cultures. Here, we propose that they may also be effective against spreading depression. We found that administration of IFNγ-DC-Exos reduced susceptibility to spreading depression *in vivo* and *in vitro*, suggesting that IFNγ-DC-Exos may be a potential therapeutic for migraine.

## Introduction

Migraine is a neurological disorder characterized by episodic severe and painful headaches lasting between 4 and 72 h. In one-third of migraine patients, headaches are preceded by focal neurological symptoms. Spreading depression (SD) is the most likely cause of migraine aura and perhaps related headache pain (Leao, 1944; Milner, 1958; Moskowitz et al., 1993; Noseda and Burstein, 2013; Pietrobon and Moskowitz, 2013). Production of the pro-inflammatory cytokine tumor necrosis factor alpha (TNFα) is increased following SD (Kunkler et al., 2004). TNFα enhances synaptic efficacy by increasing membrane expression of excitatory α-amino-3-hydroxy-5-methyl-4-isoxazolepropionic acid receptors and decreasing membrane expression of inhibitory γ-aminobutyric acid-A receptors (Stellwagen et al., 2005). Increased neuronal excitability in turn leads to increased production of reactive oxygen species, both of which promote subsequent occurrence of SD (Grinberg et al., 2012, 2013). This feedback cycle may be involved in the transformation of low-frequency migraine to higher-frequency and chronic migraine.

Our lab studies the mechanisms underlying environmental enrichment-based neuroprotection. Environmental enrichment (EE) consists of exposure to increased physical, intellectual and social activity, and has wide-ranging physiological and behavioral effects, including enhancing cognition, memory, learning, behavior and motor coordination (van Praag et al., 2000). Although the majority of EE studies have been conducted in rodents, it has been determined that EE is effective in a wide variety of animals, including non-human primates (Singhal et al., 2014). To date, these animal studies have shown that EE has beneficial effects in neurological diseases such as Huntington’s disease (van Dellen et al., 2000), Alzheimer’s disease (Jankowsky et al., 2005), and traumatic brain injury (Passineau et al., 2001). While it is more difficult to determine the effect of a robust EE paradigm in human patients, there is significant evidence that EE (which in humans includes “creative thought”) is likewise beneficial in humans.

Exposure to increased physical exercise is an important component of EE, and has been linked to improved outcomes in several neurological disorders, including depression (Strohle et al., 2010), schizophrenia (Beebe et al., 2005), epilepsy (Arida et al., 2010) and migraine (Darabaneanu et al., 2011; Varkey et al., 2011; Irby et al., 2016; Lemmens et al., 2019). Engaging in increased intellectual activity, another component of EE, is thought to play a role in creating a “cognitive reserve” that lessens the impact of brain diseases on cognitive impairment (Stern, 2012; Crescentini et al., 2014). Likewise, numerous studies report that social engagement and an active lifestyle can protect against dementia (Fratiglioni et al., 2004). In addition, EE has well-documented effects on the immune system. Much of this work has been conducted in the context of immune (dys)function with age or in response to infection. This is important, as age is a critical factor in the progression of many neurological diseases (Mattson et al., 2002; Sim et al., 2002). Despite the many benefits of EE, clinical implementation may be difficult. As a result, we have studied the signaling involved in EE-based neuroprotection with the goal of developing effective mimetics as an alternative.

We first focused on exosomes derived from the serum of EE-exposed rats. When applied to hippocampal slice cultures or nasally administered to naïve rats, these exosomes significantly increase myelin content, oligodendrocyte precursor cell and neural stem cell levels, and reduce oxidative stress (Pusic and Kraig, 2014). We next used rat bone marrow-derived dendritic cells (DCs) as a scalable, exogenous source of similarly pro-myelinating exosomes. Primary rat DC cultures were stimulated with low-level interferon gamma (IFNγ), a pro-inflammatory cytokine that phasically increases during EE. Exosomes released by IFNγ-stimulated DCs (IFNγ-DC-Exos) also increase myelination and oxidative tolerance *in vitro* and *in vivo* (Pusic A. D. et al., 2014).

Since EE has been clinically shown to reduce migraine frequency (Darabaneanu et al., 2011; Varkey et al., 2011), we also explored the mechanism of EE-mediated mitigation of SD. We found that exposure to an EE paradigm significantly reduces SD susceptibility in rats (Pusic K. M. et al., 2014). Mimicking the cytokine signaling of EE through administration of interleukin-11, insulin-like growth factor-1 or phasic administration of IFNγ likewise reduced susceptibility to SD (Pusic K. M. et al., 2014; Pusic and Kraig, 2015; Grinberg et al., 2017).

Based on the body of work outlined above, work presented here takes the next logical step to determine whether EE mimetic IFNγ-DC-Exos are also protective against SD. Treatment with IFNγ-DC-Exos reduced susceptibility to SD in hippocampal slice cultures. When nasally administered to rats, IFNγ-DC-Exos reduced susceptibility to SD, promoted a reduction in microglial M1 product iNOS, and reduced oxidative-stress mediated damage. These results provide the first evidence that IFNγ-DC-Exos, a naturally occurring biologic, is effective against SD. Accordingly, further study of this biologic as a potential therapeutic for migraine is warranted.

## Methods

### Animal Care

Wistar rats were obtained from Charles River Laboratories (Wilmington, MA, United States) and were used in accordance with the University of Chicago Animal Care and Use Committee. Untimed pregnant Wistar female rats were single-housed with Enviro-dri paper bedding (Shepherd, Watertown, TN, United States) and Nestlets (Ancare Corporation, Bellmore, NY, United States) and pups (culled to ten at birth) were used for hippocampal slice cultures. Male Wistar rats (10–12 weeks old) were double-housed and used for bone marrow isolations.

### Slice Culture Preparation and Use

P9–P10 rat pups were used to make hippocampal slice cultures (350 μm) as previously described (Kunkler and Kraig, 1997). After 18 days *in vitro* (DIV) cultures were transferred to a serum-free medium, which does not activate microglia and does not contain horse serum-derived exosomes, thus allowing for accurate assessment of the impact of exosome treatments (Pusic and Kraig, 2014). Cultures were used when mature, at 21–35 DIV. All cultures were screened for viability by staining with Sytox (Invitrogen, Carlsbad, CA, United States), a fluorescent cell death marker. Cultures with any evidence of pyramidal cell layer death were excluded.

Exosome treatments were applied to the media of slice cultures and incubated for 3 days. Treatments consisted of 100 μg of exosomes in 50 μL. All experimental measurements were compared to age-matched control slice cultures.

Slice culture electrophysiology was performed as previously described (for details see Pusic et al., 2011). Briefly, a hippocampal slice culture insert was placed in a 35 mm culture dish filled with 1.5 mL of serum-free culture medium and secured in place. A sterile cotton strip saturated in medium was placed along the inner wall of the insert to provide necessary humidity. Next, the insert-dish assembly sealed with polyvinyl chloride wrap (Thermo Fisher Scientific, Waltham, MA, United States) and placed into a recording chamber (PDMI-2; Harvard Apparatus, Holliston, MA, United States) that maintains temperature at 36°C and 5% carbon dioxide, 95% air. Recording microelectrodes and a specially fabricated bipolar stimulating electrode were positioned into slice cultures using WR 60 manipulators (Narishige International, Amytiville, NY, United States) on an inverted microscope stage (DMIRBE; Leica, Wetzlar, Germany).

Interstitial DC recordings were made using an Axoprobe A1 amplifier system coupled to a Digidata 1422A analog-digital conversion board (Axon Instruments, Burlington, CA, United States). Bipolar electrical stimuli were provided via a digital Master-8 stimulator (A.M.P. Instruments, Jerusalem, Israel) coupled to a model BSI-2 isolator (Bak Electronics, Inc., Umatilla, FL, United States). To determine SD threshold, stimulation [10 pulses, 10 Hz (100 μs/pulse)] was applied at half the current required for eliciting maximal field potential, and increased (every 3 min) until SD was induced.

### Isolation of Dendritic Cells

Immature bone marrow cells were isolated from Wistar rats, as previously described (Powell et al., 2003; Pusic A. D. et al., 2014). Briefly, animals were anesthetized with progressive exposure to 100% carbon dioxide and then immediately decapitated. Using aseptic techniques, bone marrow was aspirated out of the femurs and tibias and stromal cells were purified through the passage of bone and debris through a strainer. Red blood cell lysis buffer (0.15 M NH_4_Cl, 10 mM KHCO_3_, and 0.1 mM EDTA) was used to remove the red blood cells. Cells were then washed and plated in 6-well plates at a density of 10^6^ cells/mL in RPMI 1640 (Invitrogen) containing 10% FBS (Invitrogen) and 20 ng/mL of GM-CSF (PeproTech Inc., Rocky Hill, NJ, United States) for differentiation into bone marrow derived DCs. Media was changed on day two and five. DCs in suspension were harvested on day seven and transferred to new plates.

### Generation and Isolation of Dendritic Cell-Derived Exosomes

For generation of DC exosomes, media was prepared using 10% exosome-depleted FBS (System Biosciences, Palo Alto, CA). Day seven bone marrow dendritic cells were plated at 10^6^ cells/mL and placed in media alone or stimulated with media containing 500U of IFNγ (R&D Systems, Minneapolis, MN, United States). Three days later, culture media was collected and spun down to remove any cells and debris. Exosomes were then isolated using ExoQuick (System Biosciences). ExoQuick was added to culture media at a ratio of 1:5, incubated at 4°C overnight, and exosomes were precipitated by centrifugation at 2000×*g* for 30 min. The exosome pellet was resuspended in 100 μL of sterile phosphate buffered saline at a pH of 7.3. Isolation of exosomes was confirmed via immunoblot for two exosomal protein markers, CD63 and Alix (AbD Serotec, Kidlington, United Kingdom) (Schorey and Bhatnagar, 2008) and electron microscopy (Thery et al., 2006) (data not shown). Quantification of exosomes was performed by BCA assay (Thermo Fisher Scientific) of protein content.

### Intranasal Administration of IFNγ-DC-Exos

Wistar rats were nasally administered exosome preparations as previously described (Pusic A. D. et al., 2014). Briefly, rats were placed in a fume hood with a heat lamp and thermo-regulator to maintain temperatures at 37°C. Isoflurane (Butler Schein Animal Health, Dublin, OH, United States) anesthesia was delivered via a nose cone (five percent induction and two-three percent maintenance, delivered in oxygen). Animals were placed in a supine position, and 100 μg of exosomes in 50 μL were administered over a 20 min period at a rate of 5 μL every 2 min to alternating nostrils (Liu et al., 2001). Sham animals were administered 50 μL of sodium succinate vehicle alone, following the protocol above.

One or three days later, animals were anesthetized with progressive exposure to 100% carbon dioxide and decapitated. Brains were rapidly removed, flash frozen in isopentane, and stored at −80°C until further use.

### Whole Animal Electrophysiology

Whole animal SD recordings were completed using aseptic techniques (Kraig et al., 1991; Pusic K. M. et al., 2014). Male Wistar (300–400 gm) rats were anesthetized with isoflurane in oxygen (five percent induction, three percent during surgical procedures, two-three percent during recordings) via inhalational mask with outflow gas exhausted via vacuum to prevent room contamination. Arterial oxygen was monitored throughout with an oximeter (Nonin Medical, Plymouth, MN, United States) and ranged from 95 to 100%. Animals were continuously monitored for uniform respiratory rate and depth of respiration as well as animal color and periodic absence of withdrawal to hind paw pinch (National Research Council, 2011; Tremoleda et al., 2012).

Once anesthetized, animals were mounted in a standard table-top nose clamp and ear bars and kept warm with an overhead infrared lamp to keep core temperature at 37°C in preparation for cranial surgery. Eyes were coated with Artificial Tears (Akorn, Lake Forest, IL, United States) and the head was shaved and cleansed with Betadine (Purdue Products L.P., Stamford, CT, United States). Next, 0.05 mL of 0.25% Bupivacaine (Hospira Inc., Lake Forest, IL, United States) was injected subcutaneously to either side of what would become a midline scalp incision minutes later. A midline scalp incision was made from just behind the eyes to the lambdoid suture area. The skin was spread laterally and skull scraped free of connective tissue. Skull hemostasis was achieved using Bone Wax (CP Medical Inc., Portland, OR, United States). Two 1–2 mm craniotomies were made in the left skull under saline cooling and without damaging the underlying dura. The KCl stimulation craniotomy was placed −2.0 mm from bregma and 1.5 mm to the left of the sagittal suture. The recording craniotomy was placed −6.0 mm from bregma and 4.5 mm lateral to the sagittal suture.

After craniotomy surgery, anesthetized animals were quickly transferred to a stereotaxic recording setup where gaseous anesthesia, oxygen monitoring, and warming was continued. The skull was warmed (37°C) directly with sterile saline superfusion. For interstitial DC recordings, a 2–4 μm tip microelectrode was positioned 750 μm below the pial surface at the posterior craniotomy with a Canberra micromanipulator (Narishige) and recordings begun using an Axoprobe A1 amplifier system and Digidata 1422A analog-digital conversion board run on a PC-based computer system. For KCl-induced SD threshold measurements, a microelectrode with tip broken to 8–12 μm (1.0 mm outside diameter, 0.58 mm inside diameter; Sutter Lambda SC, Novato, CA, United States) and filled with 0.5 M KCl was positioned 750 μm below the pial surface at the anterior craniotomy. Micro-injections of KCl were administered via pressure from a Picospritzer-II electronic valve system (Parker Hannifin, Hollis, NH, United States), whose injection periods were registered directly to the permanent digital recording of interstitial DC potential changes (Grinberg et al., 2017). Injections of KCl were doubled in duration every four-to-five minutes if the previous injection failed to elicit an SD. Upon successful SD induction (i.e., SD threshold), the injection electrode was pulled up and an injection of the same pressure and duration was reproduced into 3-In-ONE^TM^ light machine oil (WD-40 Company, San Diego, CA, United States) contained in a depression well microscope slide (VWR Scientific Products, Buffalo Grove, IL, United States). The injection volume was calculated by measuring the injection sphere diameter in oil with a calibrated eye-piece micrometer on a compound microscope. As previously noted (Grinberg et al., 2017), injection volumes measured from injection into machine oil likely do not fully reflect injections made *in vivo* (Nicholson, 2001). However, any discrepancies would not be consequential as they would be a systemic error that would be equally applied to all experimental conditions.

### Protein Carbonyl Measurement

Protein carbonyl levels were measured utilizing the Protein Carbonyl Content Assay Kit (Abcam, Cambridge, MA, United States) according to manufacturer’s protocol. Briefly, protein was extracted from the neocortex of animals nasally administered IFNγ-DC-Exos or sodium succinate vehicle (sham) using RIPA buffer. Protein homogenate was treated with streptozocin to remove any nucleic acid contaminates. Samples were reacted with 2, 4-Dinitrophenylhydrazine followed by quantification of the acid hydrazones at 405 nm. BCA assays (Thermo Fisher Scientific) were simultaneously run and a standard curve constructed for the calculation of protein carbonyl content based on optical density.

### RT-qPCR

RNA was isolated by TRIzol extraction followed by miRNeasy mini kit (Qiagen, Hilden, Germany) spin column-based purification. Total RNA concentrations were determined using a Take3 Micro-Volume plate, read in a Synergy HTX multi-mode reader (BioTek Instruments, Winooski, VT, United States). Equal amounts of RNA for each sample were reverse transcribed in a T100 thermocycler (Bio-Rad, Hercules, CA, United States) using the iScript cDNA synthesis kit (Bio-Rad) following manufacturer’s protocol. Real-time PCR reactions were performed using iQ SYBR Green Supermix (Bio-Rad) on the CFX96 Real Time PCR Detection System (Bio-Rad). All primers (see Table 1) were used at 10 nM (Integrated DNA Technologies, Inc., Coralville, IA, United States). Each sample was normalized to an endogenous control, Rpl13a, and the fold changes for each gene assayed was determined via the delta Ct method (Pfaffl, 2001).

**TABLE 1:** RT-qPCR Primer Sequences.

**Gene**	**GenBank Accession #**	**Primer**	**Sequence (5′–3′)**
TNFα	NM_012675.3	F R	ACCACGCTCTTCTGTCTACTGA CTGATGAGAGGGAGCCCATTTG
IL-11	NM_133519.4	F R	GCTGACAAGGCTTCGAGTAG TCTTTAGGGAAGGACCAGCT
CD32	NM_175756.1	F R	CCAAACTCGGAGAGAAGCCT CTTCGGAAGACCTGCATGAGA
CD86	NM_020081.1	F R	GAGCTCTCAGTGATCGCCAA CAAACTGGGGCTGCGAAAAA
Arg-1	NM_017134.3	F R	TGGACCCTGGGGAACACTAT GTAGCCGGGGTGAATACTGG
CD206	NM_001106123.2	F R	AGTCTGCCTTAACCTGGCAC AGGCACATCACTTTCCGAGG
iNOS	NM_012611.3	F R	AGAGACGCTTCTGAGGTTCC GTTGTTGGGCTGGGAATAGC
TGFβ	NM_031131.1	F R	ACCGCAACAACGCAATCTATG TTCCGTCTCCTTGGTTCAGC
IL-10	NM_012854.2	F R	GCTCAGCACTGCTATGTTGC AATCGATGACAGCGTCGCA
Rpl13α	NM_173340.2	F R	TTGCTTACCTGGGGCGTCT CCTTTTCCTTCCGTTTCTCCTC

### Data Handling and Statistics

All data were analyzed using SigmaStat software (Systat Software Inc., San Jose, CA, United States). All data were subject to normality testing (*p*-value to reject: 0.05). Controls in each treatment group were scaled to 1.0 with experimental data scaled proportionally and expressed as mean ± standard error of the mean. Statistical tests are noted in the figure legends.

## Results

### IFNγ-DC-Exos Increased SD Threshold in Slice Cultures

**FIGURE 1:**
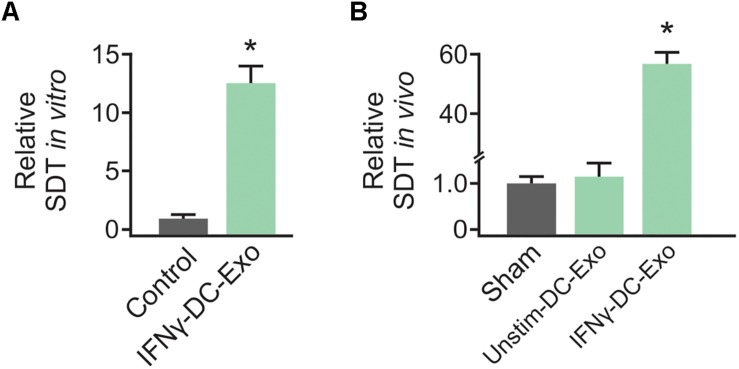
IFNγ-DC-Exosomes increase spreading depression threshold *in vitro* and *in vivo.* **(A)** When applied to naïve hippocampal slice cultures, IFNγ-stimulated dendritic cell exosomes (IFNγ-DC-Exos) significantly (*^∗^p* ≤ 0.001) increased spreading depression threshold three days later, compared to untreated control slices. **(B)** Nasally administering IFNγ-DC-Exos to rats likewise significantly (*^∗^p* ≤ 0.001) increased the threshold to neocortical spreading depression one day later when compared to untreated sham animals, or animals nasally administered unstimulated dendritic cell exosomes (Unstim-DC-Exos). Significance determined by ANOVA plus *post hoc* Holm-Sidak testing.

Preliminary work to determine the efficacy of IFNγ-DC-Exos at increasing SD threshold was done in hippocampal slice cultures. Slice cultures were treated with 100 μg of IFNγ-DC-Exos (as before, see Pusic A. D. et al., 2014) or left untreated (Control) and SD threshold measured three days later. Treatment with IFNγ-DC-Exos evoked a significant, greater than 12-fold increase in SD threshold compared to untreated control slices. Specific values were: Control: 1.00 ± 0.45; IFNγ-DC-Exos: 12.5 ± 1.52 (*n* = 8/group) (Figure 1A). This allowed us to proceed to *in vivo* experiments.

### Nasal Administration of IFNγ-DC-Exos Increased SD Threshold

Male Wistar rats were nasally administered IFNγ-DC-Exos (100 μg in 50 μL), unstimulated dendritic cell exosomes (unstim-DC-Exos) or vehicle alone (control/sham) and SD threshold determined one day later. IFNγ-DC-Exo treatment significantly increased threshold to SD when compared to age and treatment-matched sham and age-matched untreated control animals. Specific values were: Sham: 1.00 ± 0.14; unstim-DC-Exo: 1.10 ± 0.28; IFNγ-DC-Exos: 57.0 ± 4.27 (*n* = 3–5/group) (Figure 1B).

### Nasal Administration of IFNγ-DC-Exos Altered Microglial Polarization

**FIGURE 2:**
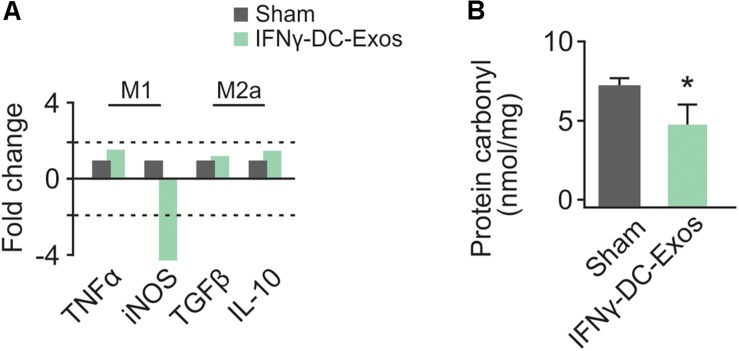
Nasal administration of IFNγ-DC-Exosomes reduces oxidative stress. **(A)** Nasal administration of IFNγ-DC-Exos significantly reduced expression of the M1 polarization product iNOS by 4.37-fold compared to expression levels in Sham animals. Significance was determined by greater than 2-fold change. Though there was no significant change in other M1 and M2a markers measured, this reduction in iNOS was reflected by a similarly significant (^∗^*p* = 0.035) reduction in **(B)** protein carbonyl content in Sham versus IFNγ-DC-Exo-treated animals. Significance determined by ANOVA plus *post hoc* Holm-Sidak testing.

As no significant differences have been observed in unstim-DC-Exo treated versus sodium succinate-treated animals thus far, we proceeded with IFNγ-DC-Exos versus sodium succinate (Sham) for subsequent experiments. Male Wistar rats were once again nasally administered IFNγ-DC-Exos or sodium succinate. One day later, brains were harvested and RT-qPCR was performed for M1 and M2a gene expression. M1 polarization was determined by measuring expression levels of M1 products TNFα and iNOS. Similarly, M2a polarization was determined by measuring expression levels of M2a products TGFβ and IL-10. A significant decrease (4.37-fold) was found in iNOS mRNA expression in IFNγ-DC-Exo- treated brains versus untreated brains, though no significant change (<2-fold) was seen in M1-specific TNFα or M2a-specific TGFβ and IL-10 mRNA levels (Figure 2A). This may indicate that IFNγ-DC-Exo treatment reduces M1 microglial polarization, as microglia are a primary source of iNOS in the CNS (Dringen, 2005).

### Nasal Administration of IFNγ-DC-Exos Reduced Protein Carbonyl Content

Neocortical tissue was collected for determination of protein carbonyl content three days after nasal administration. Although protein carbonylation is reactive oxygen species-mediated and does not encompass reactive nitrogen species-mediated damage, its measurement is accepted as a reliable indicator for the extent of oxidative damage (Ghezzi and Bonetto, 2003). Relative to sham animals administered vehicle alone, IFNγ-DC-Exo-treated rat brains contained significantly reduced baseline levels of carbonylated protein [Sham: 7.25 ± 0.83 (*n* = 7); IFNγ-DC-Exos: 4.58 ± 0.55 (*n* = 5)] (Figure 2B).

## Discussion

This study is based on the premise that EE has great potential as a therapeutic for a wide range of neurological disorders, including migraine. A large body of literature provides evidence suggesting that EE has a beneficial impact on human brain health. Unfortunately, it can be prohibitively difficult to implement a robust EE paradigm in patients. As a result, there is increased interest in development of effective EE-mimetics (McOmish and Hannan, 2007). To this end, our lab studies the role of exosomes in EE and the use of dendritic cells as a scalable *ex vivo* source of EE-mimetic exosomes.

In prior work, we have shown that IFNγ-stimulated dendritic cell-derived exosomes reduce oxidative stress and improve recovery from a demyelinating injury in slice cultures (Pusic A. D. et al., 2014). We have also illustrated a link between myelin integrity and susceptibility to SD (Pusic et al., 2015). This led us to explore the ability of IFNγ-DC-Exos to mitigate SD as a model of migraine with aura.

Here, we demonstrate that IFNγ-DC-Exos indeed protect against SD. The ∼12-fold increase in SD threshold in slice cultures was modest, but encouraging. In the *in vivo* condition, IFNγ-DC-Exos produced a much more robust, 57-fold increase in SD threshold. While this is roughly equivalent to the protection seen with nasally administered IFNγ (∼63-fold; Pusic and Kraig, 2015), administration of IFNγ-DC-Exos does not have the same potential for negative side effects as use of a pro-inflammatory cytokine does.

Crosstalk between inflammation and oxidative stress is likely involved in SD. In prior work, we have shown that microglial polarization state is a key factor in determining SD susceptibility. Microglia, like macrophages, can be polarized by cytokines and other factors in the microenvironment to adopt anti- or pro-inflammatory phenotypes with distinct functions. These phenotypic types range from a classically activated (M1) state to an alternatively activated (M2a) state (Ransohoff and Perry, 2009). It is important to note that these polarization states are plastic, and the classifications designated here represent extremes along a spectrum of phenotypes.

Spreading depression creates an M1-skewed microglial phenotype that may increase susceptibility to subsequent SD (Pusic K. M. et al., 2014). M1 microglia produce reactive oxygen species and pro-inflammatory cytokines, including TNFα which can increase neuronal excitability and promote initiation of SD (Durafourt et al., 2012; Grinberg et al., 2012, 2013; Ajmone-Cat et al., 2013). This pro-inflammatory environment may also be responsible for transient gray matter myelin disruption following SD (Pusic et al., 2015). Conditions of increased inflammation and oxidative stress deplete intracellular glutathione levels and activate neutral sphingomyelinase-2 (Liu et al., 1998; Jana and Pahan, 2007). Myelin contains neutral sphingomyelinases (Chakraborty et al., 1997) whose activity leads to sphingomyelin hydrolysis, ceramide formation and other downstream reactions with deleterious effects on myelin integrity. Given that demyelination in the cuprizone model of MS increases susceptibility to SD (Hoffmann et al., 2008), we suggest that SD-induced demyelination may also contribute to increased SD susceptibility by enhancing aberrant excitability, via ephaptic transmission between demyelinated fibers.

Nasal administration of IFNγ-DC-Exos reduced levels of iNOS mRNA (a product of M1 polarization) and decreased protein carbonyl content, suggesting that IFNγ-DC-Exos create an environment of decreased oxidative stress. This conclusion is supported by prior work demonstrating that IFNγ-DC-Exo treatment reduces menadione-induced oxidative stress and increases microglial glutathione in hippocampal slice cultures (Pusic A. D. et al., 2014). While we acknowledge that other neuronal cell types produce iNOS, microglia are a major cellular source in the CNS (Dringen, 2005). Furthermore, we have previously shown that rats exposed to EE have significantly lower M1 and higher M2a levels than non-enriched animals, and found evidence that EE protection against SD is in part mediated by decreased generation of oxidants and pro-inflammatory cytokines from M1 microglia (Pusic K. M. et al., 2014).

Finally, we have demonstrated in several instances (Pusic and Kraig, 2014; Pusic A. D. et al., 2014) that exosomes can be nasally administered to impact brain function. Here, we have extended those studies to show that nasal administration of exosomes can effectively decrease susceptibility to SD in an *in vivo* model of migraine. While we can infer that exosomes enter the CNS through measurement of these functional effects, we have not yet directly tracked exosomes through imaging studies. Future studies should also involve tracking exosomes post-nasal administration to whole animals to determine the efficiency of this delivery route to the brain, the rate of entry and clearance, and cellular uptake.

In conclusion, work here discusses several mechanisms by which exosome-based neuroimmune signaling contributes to neuroprotection resulting from EE, and advocates use of EE-mimetic exosomes as neurotherapeutics.

## Data Availability

The datasets generated for this study are available on request to the corresponding author.

## Ethics Statement

Animal Subjects: The animal study was reviewed and approved by The University of Chicago Animal Care and Use Committee.

## Author Contributions

AP and KP conceived the study and wrote the manuscript. All authors performed the experiments and analyzed the data, read and commented on the final version of the manuscript and approved the submission.

## Conflict of Interest Statement

AP, KP, and RK are co-inventors listed on the following issued and pending patent applications: United States Patent No. 10, 231,997 issued March 19, 2019; United States Patent Application No. 16/259, 563 filed January 28, 2019; Canadian Patent Application No. CA2882248 filed August 15, 2013, European Patent Application No. 13829748.6 filed August 15, 2013; and Australian Patent No. AU2013302526 issued July 5, 2018; all dealing with the use of exosomes to reduce oxidative stress in the central nervous system and promote remyelination of damaged neurons. The remaining author declares that the research was conducted in the absence of any commercial or financial relationships that could be construed as a potential conflict of interest.
